# Green carbon science: fundamental aspects

**DOI:** 10.1093/nsr/nwad046

**Published:** 2023-02-24

**Authors:** Mingyuan He, Kun Zhang, Yejun Guan, Yuhan Sun, Buxing Han

**Affiliations:** Shanghai Key Laboratory of Green Chemistry and Chemical Processes, School of Chemistry and Molecular Engineering, East China Normal University, Shanghai 200062, China; Research Institute of Petrochem Processing, SINOPEC, Beijing 100083, China; Institute of Eco-Chongming, Shanghai 202162, China; Shanghai Key Laboratory of Green Chemistry and Chemical Processes, School of Chemistry and Molecular Engineering, East China Normal University, Shanghai 200062, China; Institute of Eco-Chongming, Shanghai 202162, China; Shanghai Key Laboratory of Green Chemistry and Chemical Processes, School of Chemistry and Molecular Engineering, East China Normal University, Shanghai 200062, China; Institute of Eco-Chongming, Shanghai 202162, China; Low Carbon Energy Conversion Center, Shanghai Advanced Research Institute, Chinese Academy of Sciences, Shanghai 201203, China; Shanghai Low Carbon Technology Innovation Platform, Shanghai 210620, China; Shanghai Key Laboratory of Green Chemistry and Chemical Processes, School of Chemistry and Molecular Engineering, East China Normal University, Shanghai 200062, China; Beijing National Laboratory for Molecular Sciences, Institute of Chemistry, Chinese Academy of Sciences, Beijing 100190, China; Institute of Eco-Chongming, Shanghai 202162, China

**Keywords:** green carbon science, carbon cycle, carbon energy, carbon neutrality, redox chemistry

## Abstract

Carbon energy has contributed to the creation of human civilization, and it can be considered that the configuration of the carbon energy system is one of the important laws that govern the operation of everything in the universe. The core of the carbon energy system is the opposition and unity of two aspects: oxidation and reduction. The operation of oxidation and reduction is based on the ternary elemental system composed of the three elements of carbon, hydrogen and oxygen. Its operation produces numerous reactions and reaction products. Ancient Chinese philosophy helps us to understand in depth the essence of green carbon science, to explore its scientific basis, and to identify the related platforms for technology development.

## Teaser

There is no life without carbon and no life can survive without carbon dioxide. The key factors are balance and recycling. The essence in chemistry for carbon energy system and carbon neutrality is the opposition and unity of two aspects: oxidation and reduction.

The concept of green carbon science was proposed with the purpose of deepening our understanding of the issues related to carbon, based on the whole process of carbon resource processing, carbon utilization, and carbon recycling, and to attempt seeking scientific and technological solutions for achieving the goal of carbon neutrality [1–4].

**Figure 1.**
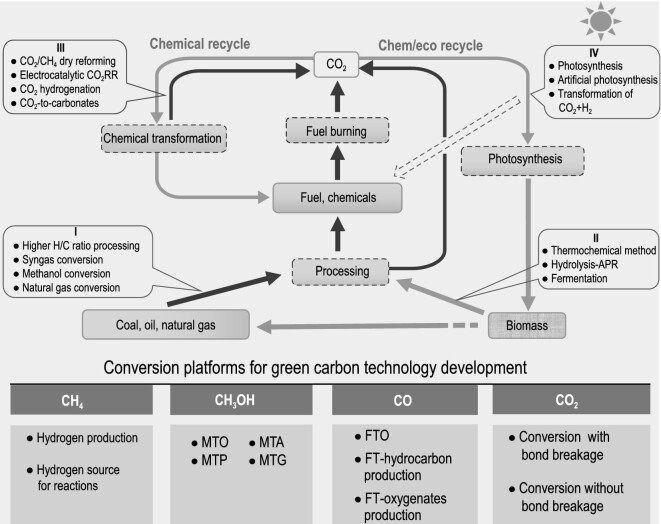
Schematic diagram of simplified carbon cycle related to the utilization of fossil resources, conversion of biomass, and recycling of CO_2_. Four phases are covered by the scheme of carbon recycling, and four platforms are illustrated for the practice of green carbon technology development focusing on carbon neutrality.

The carbon energy system will be, substantially, a carbon cycle system. Figure 1 illustrates four phases of transformation reactions in the functioning of the operation of the carbon energy system, as well as four platforms for the development of practical technological solutions focusing on carbon neutrality. The black and green arrows following before or after the CO_2_ box, indicate the direction to oxidation or reduction, respectively. In short, oxidation and reduction are essentially the two opposite and unified halves of the carbon energy system.

Generating energy from carbon, and simultaneously releasing carbon dioxide, is a process that has taken place for as long as the Earth has had organisms living on it. The advent of human beings has added to the increase of carbon dioxide emissions by the combustion of carbon-containing substances to obtain thermal energy, and further to acquire kinetic energy and electric energy, *etc.* Especially because of the Industrial Revolution, carbon dioxide emissions have increased dramatically, and the carbon balance that could be maintained before no longer exists. Consequently, people are actively searching for renewable energy. Renewable energy is more than just biomass and carbon-free energy; carbon dioxide can also be considered a green chemical when it is utilized as renewable energy. Carbon dioxide usage, derived by renewable energy, thus contributes directly to carbon neutrality.

**Figure 2.**
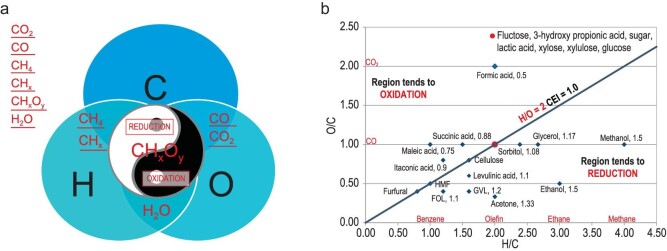
(a) One carbon energy system, comprises two opposite and unified halves, oxidation and reduction depicted as Taijitu, and three elements carbon, hydrogen, and oxygen interacting with each other to accomplish all oxidation-reduction reactions. The molecules listed on the left side are primary products of the ternary elemental system. (b) A coordinate system shows, again, a one carbon energy system, two regions, and ternary elemental compositions.

Avelino Corma [5] pointed out that ‘Carbon is indispensable—there is no life without carbon and no life can survive without carbon dioxide. In short, the pivotal factors for carbon are balance and recycling’. One of the innermost books of ancient philosophy ever written is ‘Tao Te Ching’ by Lao Tzu (571–471 B. C.). It is said that the *Tao Te Ching* ranks with the Bible as one of the most translated books of all time. One of the English versions was translated by Derek Lin [6]. In the book, Chapter 42 says that: ‘Tao produces one. One produces two. Two produce three. Three produce myriad things. Myriad things, backed by Yin and embracing Yang achieve harmony by integrating their energy.’ Here, as a simplified common understanding, ‘Tao’ means the universe and its laws. Following ‘Tao’, ‘One’ means carbon energy as the object we are studying in green carbon science. A common theme in Taoism is the duality of all things in nature, visually depicted as the symbol shown in the core part of Fig. 2a. The symbol has a Chinese name of Taijitu. The white region represents Yin, or Negative, and the black region represents Yang, or Positive. Two complementary halves play against each other at all times and one cannot exist without the other. Figure 2 illustrates that, when the carbon energy system discussed is one, the core of the system being composed of two opposite counterparts, oxidation and reduction, can be taken as two, and the three elements, carbon, hydrogen and oxygen, constituting the carbon energy system, and effectively accomplishing all oxidation-reduction reactions, can be taken as three. Based on the three elements of carbon, hydrogen and oxygen, many reactions can be generated and many molecules can be produced, as shown on the left side of the figure for six primary products. That means three produces myriad things. The molecules produced may further react with each other to derive a series of new reactions and a variety of new products.

We have recently proposed the concept of a carbon energy index (CEI) [2], which is associated with the number of C, H, and O in the molecules. The calculated carbon energy index (indicated after the name of the compound) is used to map or locate the molecules in the oxidation-reduction coordinate system. The oblique line (H/O = 2, CEI = 1) divides all molecules into two regions. The CEI value of all molecules in the upper region is <1, which can be considered as the region tends toward oxidation. All molecules in the lower region have CEI values >1, which can be considered as the region tends toward reduction. As shown in Fig. 2, many familiar platform molecules for biomass conversion are located in the lower region of the coordinate system.

**Figure 3.**
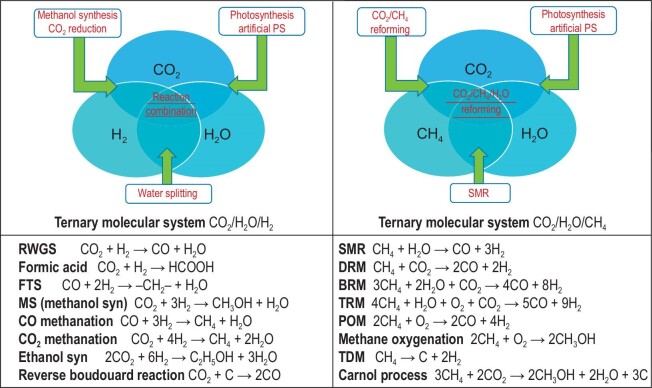
Ternary molecular systems are composed of three molecules, CO_2_, H_2_O, H_2_, and CO_2_, H_2_O, CH_4_. The reactions between molecules in each system are listed in the lower part. RWGS—reverse water gas shift; FTS—Fischer-Tropsch synthesis; SMR—steam methane reforming; DRM—dry reforming of methane; BRM [7]—bi-reforming of methane; TRM [7]—tri-reforming of methane; POM—partial oxidation of methane; TDM [8,9]—thermal decomposition of methane. The Carnol process [8,9] comprises the reactions of thermal decomposition of methane and methanol synthesis from CO_2_.

The carbon energy system is based on the ternary elemental system of carbon, hydrogen and oxygen, with six molecules CO_2_, CO, CH_4_, CH_x_, CH_x_O_y_, and H_2_O, as primary products closely related to the system. Starting from the six product molecules, further reactions have been explored to satisfy the social demand for fuels and chemicals. Obviously, the transformation of CO_2_ is the key factor for developing a carbon cycle system. The reduction of CO_2_ can be realized by acquiring hydrogen atoms from H_2_, CH_4_, or H_2_O. Therefore, ternary molecular systems can be used to systematically interpret the interrelated reactions. For instance, one is composed of carbon dioxide, water, and hydrogen, the other is composed of carbon dioxide, water, and methane, as shown in Fig. 3. The reactions between molecules of each ternary molecular system are also listed in the lower part of the figure. A variety of reactions and molecules can be further developed. The figure could be of help in understanding some aspects of the research frontiers of modern energy chemistry.

**Figure 4.**
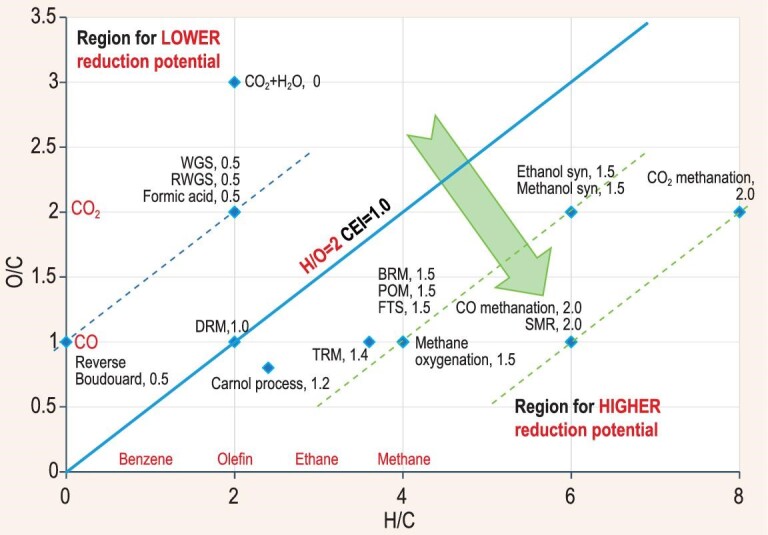
The oxidation-reduction coordinate used for mapping reduction potentials of different reaction systems. The designations used for the specific reaction systems are the same as those in Fig. 3.

The oxidation-reduction coordinate system can also be used to map the ternary molecular reactant systems of Fig. 3 to observe their potential toward reduction, as shown in Fig. 4. For each reaction listed in Fig. 3, the values of the total atom ratio of H/C and O/C can be calculated for the reactant mixture of the reaction, and used to locate the designated reaction. The sum of CEI values of the molecules in the reactant mixture are also calculated and indicated. The reactant system composed of carbon dioxide and water is located at the highest position in the upper region in the coordinate system, with a sum CEI value of zero, indicating the intrinsic reduction potential is zero. The location of reactions WGS (CO + H_2_O), RWGS (CO_2_ + H_2_), and direct formic acid synthesis (CO_2_ + H_2_) are consistent with their low reduction potential. The DRM (CO_2_ + CH_4_) reaction is located on the dividing line. In comparison, the locations of the BRM (3CH_4_ + 2H_2_O + CO_2_) reaction and TRM (4CH_4_ + H_2_O + O_2_ + CO_2_) reaction move downward to the lower region, showing better reduction potential. It is interesting to see that the location of BRM is exactly in-between DRM and SMR (CH_4_ + H_2_O) and the location of TRM is on the same horizontal line with DRM, SMR, and POM (2CH_4_ + O_2_) and at a position left of the location of POM. The Carnol process (3CH_4_ + 2CO_2_) was developed based on the combination of thermal decomposition of methane (TDM, CH_4_ → C + 2H_2_) and methanol synthesis (MS, CO_2_ + 3H_2_ → CH_3_OH + H_2_O). The reactant mixture of Carnol process and DRM are both composed of two molecules, CH_4_ and CO_2_, they are both located on the oblique line connecting the points of CH_4_ on the x-axis and CO_2_ on the y-axis. However, the lower location of Carnol process indicates higher reduction potential compared with DRM. Among all those elemental reactions listed in Fig. 3, different reaction combinations can be engineered for special purposes or for process development. In Figs 3 and 4, the reaction combinations can be recognized as follows:


\begin{eqnarray*} {\rm{BRM}} &=& {\rm{DRM}} + 2\ {\rm{ SMR}}\\ {\rm{TRM}} &=& {\rm{DRM}} + {\rm{SMR}} + {\rm{POM}}\\ {\rm{Carnol\ Process}} &=& 3\ {\rm{ TDM}} + {\rm{MS}} \end{eqnarray*}

**Figure 5.**
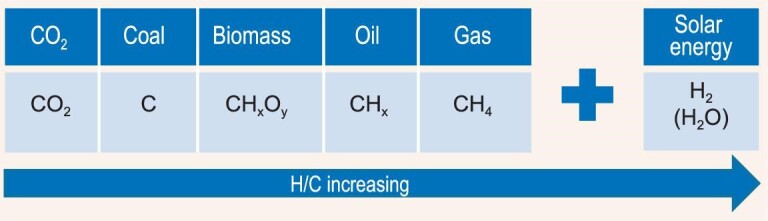
Hydrogen is the key for CO_2_ utilization and hydrogen-deficient energy resource processing.

Carbon dioxide, considered as a carbon-containing energy carrier, can be compared with fossil energy resources and biomass for their elemental composition as shown in Fig. 5. From left to right the oxygen content decreases and the hydrogen content increases. The key to the effective utilization of carbon dioxide and some hydrogen-deficient fossil energy is, obviously, hydrogen. Increased use of natural gas would certainly help mitigate the urgent requirement. The green utilization of natural gas, with high carbon atom efficiency and energy efficiency, is of significant importance, especially at the present stage. Hydrogen production technology with no or limited CO_2_ formation are urgently needed. Applying solar energy to decompose water for hydrogen production is ultimately the best choice. However, the ternary elemental system and the ternary molecular systems we discussed may provide a basis for strategic considerations in developing the modern energy industry.

Chemistry is the central science for material transformation. The underlying scientific issues of the carbon energy system are closely related to its basic elemental compositions and interrelations. The transformation between different carbon energy resources, including CO_2_, starts from the activation of reactant molecules. The different carbon active species possess different ternary elemental compositions of carbon, hydrogen, and oxygen. The composition changes of the carbon species eventually lead to either oxidation or reduction reactions within the carbon energy system. Since hydrogen is important, applying solar energy to decompose water for hydrogen production is ultimately the best choice. Water electrolysis is currently more effective than solar-driven water decomposition. Meanwhile, the approach to acquire and use hydrogen atoms directly from water molecules instead of molecular hydrogen is an attractive proposition. The key to water as a hydrogen source is the activation of water or its O–H bond at the nanoscale interface by catalyst design. Furthermore, in-depth understanding of redox chemistry is necessary for the re-construction of the carbon energy system. In fact, its essence is how to manipulate electron transfer at the molecular level, which is often accompanied by proton transfer, i.e. the concerted proton coupled electron transfer (PCET). For the redox chemistry involved in the activation and inter-transformation of reactant molecules in the ternary molecular system of CO_2_, hydrogen and water (Fig. 3), the electron transfer could not be an isolated event, but a concurrent one initiated by a catalyst.

**Figure 6.**
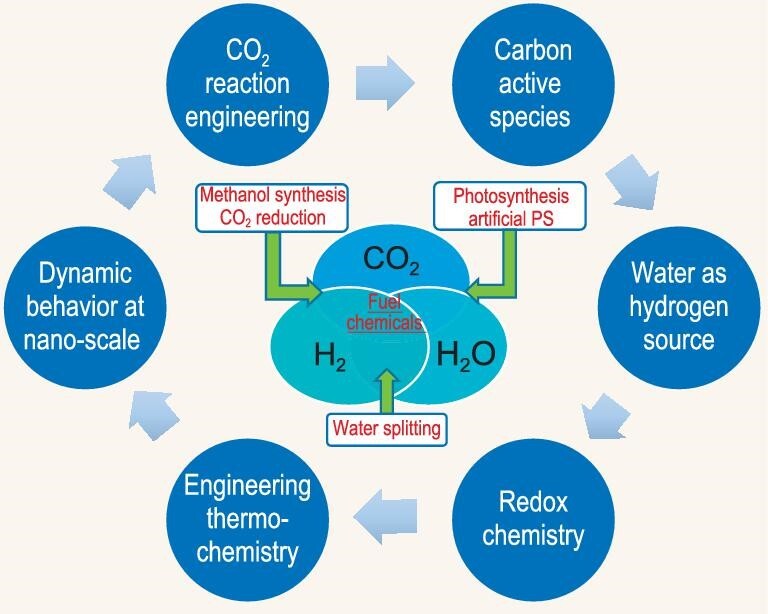
Six fundamental aspects of green carbon science that is symbolized by a tri-molecular system of CO_2_, hydrogen and water.

Figure 6 summarizes several fundamental aspects of green carbon science. The concept of green carbon science here is represented by the ternary molecular system of CO_2_, hydrogen and water, denoted in the core part of the figure, to emphasize the importance of those molecules in the current carbon energy system. The related fundamental aspects are shown around the figure, as mentioned above, starting from active carbon species, followed by water and hydrogen bonding, redox chemistry, engineering thermochemistry, dynamic behavior at the nanoscale, and finalized by CO_2_ reaction engineering. The concept of ‘engineering thermochemistry’ was discussed by Xu *et al.* [10]. For processing hydrogen-deficient carbon energy resources usually two opposite solutions exist, one is hydrogen incorporation and the other is decarbonization. The latter, in the sense of chemical transformation, is closely related to engineering thermochemistry. Moreover, technologies related to biomass and CO_2_ utilization, plastic waste, and urban waste recycling, are all heavily dependent on engineering thermochemistry. In short, the circular economy is out of the question without engineering thermochemistry. Green carbon science [1] covers the transformation of carbon-containing compounds and the relevant processes involved in the entire carbon cycle from carbon resource processing, carbon energy utilization, CO_2_ fixation, and carbon recycling. Obviously, mass transfer is an extraordinarily complex problem for the transformation system involved, which is characterized in multi-scale, multi-phase, and multi-step in nature. The significance of the dynamic behavior at the nanoscale is well demonstrated in the selective conversion of methanol in catalytic materials with different pore structures, such as SAPO-34 and ZSM-5. It implies that dynamic observation and understanding is important for acquiring a deep insight of reaction mechanisms in nanoreactors. For carbon dioxide transformation, the practical purpose of the research is to lead to the development of carbon dioxide processing processes and finally to establish a carbon dioxide industry, possibly in parallel with the coal industry, oil industry, and natural gas industry. Hence, reaction engineering studies and novel reactor inventions based on new concepts and new designs for effective CO_2_ conversion are critical, including membrane reactors, micro-channel reactors, integrated modular reactors, *etc.*

**Figure 7.**
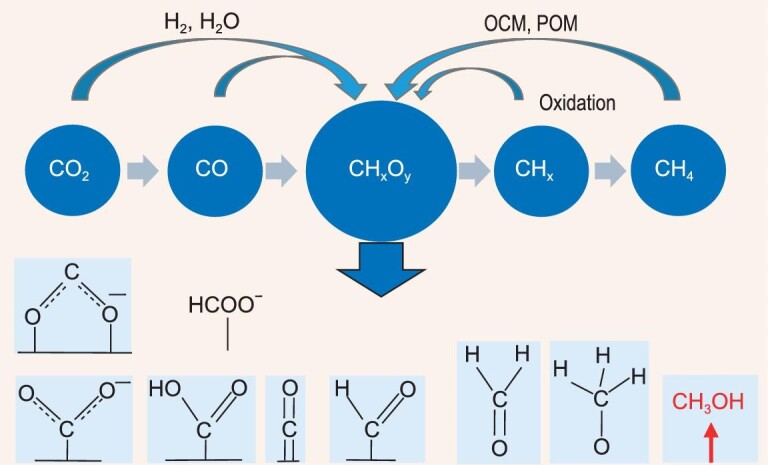
The formation of ternary elemental species CH_x_O_y_ is the key step for transformation reactions starting from either CO_2_/CO or CH_4_/CH_x_.

**Figure 8.**
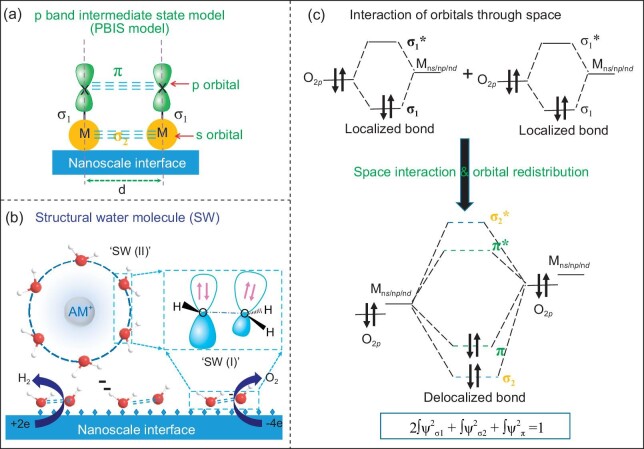
The p band intermediate state (PBIS) theory (a) was proposed to understand the unique role of structural water molecules (SWs) at confined nanoscale interface to accelerate the reaction kinetics of HER and OER as a typical example (b). The nature of PBIS is the overlap of p orbitals of heteroatoms (O/C/S/N/P) by space interactions in the confined nanospace (c), which provides some alternative channels for surface electron and proton transfer.

**Figure 9.**
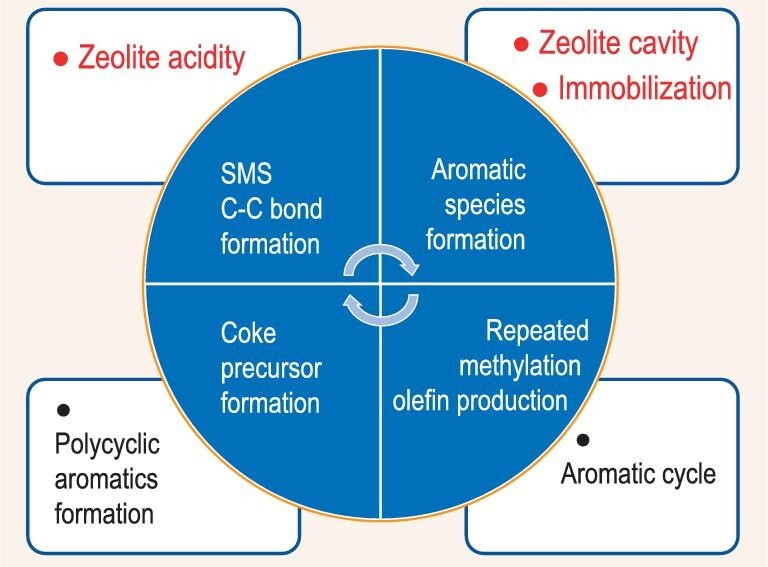
The cavity of SAPO-34 porous structure with acid sites and confined space catalyze a series of reactions as a nanoreactor for MTO processes.

- **Carbon active species.** The transformation of carbon-containing molecules begins with the activation of molecules forming carbon active species. Carbon energy containing molecules are made up of three kinds of atoms, carbon, hydrogen and oxygen, so these are the carbon active species. Carbon active species, as reaction intermediates, decide the selectivity of the transformation reactions. In heterogeneous catalysis, the formation of active carbon species depends significantly on the catalysts. Different catalyst surfaces and reaction conditions make the carbon-containing molecules form different carbon-active species, such as carbon cations, carbon anions, free radicals, free radical carbon ions, *etc.*, existing on the catalyst surface in adsorption states or in the gas phase. The composition of carbon, hydrogen, and oxygen elements and the bonding between atoms change simultaneously during the transformation of carbon active species and dictate the selectivity of the final products. Two opposite directions are shown in Fig. 7. The direction from left to right indicates that the incorporation of hydrogen, or hydrogen from water, leads to the formation of ternary elemental species CH_x_O_y_ from CO_2_ or CO. In contrast, the direction from right to left indicates that the incorporation of oxygen leads to the formation of ternary elemental species CH_x_O_y_ from CH_4_ or CH_x_ (hydrocarbon molecules). The formation of ternary elemental species is the key step for all transformation reactions of carbon energy containing molecules.
  He *et al.* [11–14] studied different adsorption states and transformations of CO and CO_2_ on a variety of metal oxides. In the presence of hydrogen, these species can eventually produce HCOOH, H_2_CO, HCOOCH_3_, CH_3_OH, and CH_4_. The formation of the adsorption state of ternary elemental species has a decisive effect on product selectivity. Liu *et al.* [15] summarized the adsorbed state of CO_2_ on the metal center and pointed out that the coordination between the metal center and carbon atoms was crucial to the formation of C–C bonds, and C-C bonds can be formed by inserting a CO_2_ molecule into the C-M bond [16]. The C-C bond formation has been a hot topic in the research area of C_1_ chemistry. It is well known that two transformation platforms have been developed in the area of C_1_ chemistry: syngas conversion platform and methanol conversion platform. However, the mechanistic study of different conversion platforms and different transformation pathways reveals a fact that the hub of the transformation rests with certain key carbon active species coordinated with hydrogen and oxygen. With CO_2_ or CO as the starting reactant, Wei *et al.* [17] proposed and confirmed that the ‘Aldol-aromatic’ mechanism is a way of hydrogenation to hydrocarbon aviation fuel. CO_2_ and hydrogen were activated on the surface of an oxide catalyst to form adsorption states of HCHO, HCOOH, and CH_3_OH, and then diffused into HZSM-5 zeolite through the surface to undergo the Aldol reaction, forming C-C bonds and subsequently aromatic rings and cyclic oxygen-containing compounds by condensation. Obviously, for the Aldol reaction, HCHO or *CHO is the key intermediate. On the other hand, with methanol as the starting reactant, Liu and Lercher *et al.*, [18] studied MTO reaction on ZSM-5 catalyst, by tracing the reaction pathways of ^13^C-labeled formaldehyde, it is shown that formaldehyde reacts with alkenes via Prins reaction into dienes and finally to aromatics. Therefore, carbon-hydrogen-oxygen ternary elemental species CH_x_O_y_ in general, is the hub of the transformation reaction from two opposite directions, and HCHO or *CHO is the key intermediate of these reactions, especially for C–C bond formation.
  Han *et al.* [19] studied electrochemical reduction of carbon dioxide using copper selenide as the catalyst which exhibited outstanding performance for electrochemical reduction of carbon dioxide to methanol. To understand the reaction pathway for the formation of methanol, some possible reaction intermediates, such as formic acid, CO and formaldehyde, were introduced in experiments, and evidenced that CO and formaldehyde clearly promoted the formation of methanol. The DFT study indicates that after the formation of adsorbed *CO species, the step of *CO reduction to *CHO was an endothermic and likely rate-limiting step. The *CHO species is easier to adsorb on the surface of the studied catalyst to accept electrons and protons to form *OCH_2_ and *OCH_3_, and is then reduced to methanol. Again, the key reaction intermediates here for the transformation from carbon dioxide to methanol is clarified to be HCHO or *CHO species [19]. In biomass conversion, a sugar-bitungstate complex was proposed to be the key intermediate for C–C breakage of sugar molecules [20], which provides a facile way of producing C_2,3_ oxygenates following a non-classical retro-aldol condensation mechanism from biomass derivatives.
- **Water as a hydrogen source.** Hydrogen is the simplest element in the ternary elemental system, but vital for the carbon energy system in CO_2_ utilization and hydrogen-deficient energy resource processing. Hydrogen is involved in the formation of ternary elemental species CH_x_O_y_ as reaction intermediates in reduction reactions. Theoretically, all the compounds containing H atoms can be H donors, such as molecular hydrogen, ammonia, borohydride, borane, alcohols, formic acid and water, *etc.* Among them, molecular hydrogen is the most commonly used and hence the construction of molecular hydrogen-based energy system and hydrogen economy has drawn great attention worldwide. Efficient green or low carbon production of hydrogen by water splitting is a target for human society. However, in the carbon energy system hydrogen atoms involved in the transformation reactions may not necessarily originate from molecular hydrogen, as shown in Figs 3 and 7. The direct use of hydrogen atoms from water molecules instead of molecular hydrogen is an attractive proposition. When water was used as a hydrogen source, the adsorption of water, the subsequent activation and scissoring of water O–H bonds, and concurrently the transfer of H atoms at the nano-scale interface are key steps. Thus, the choice of the catalyst with a finely-tuned microscale aqueous environment around active sites is essential for the formation of active hydrogen species and subsequent insertion of hydrogen.
  Several typical examples in the following show that, by the rational design of multifunctional catalysts, water can be directly used as a hydrogen source for hydrogen production or as a co-catalyst promoting the yield of hydrogen or liquid fuel. Solid experimental evidence presented by state-of-the-art *in situ* (operando) spectroscopic techniques showed that the micro-structure of water clusters and H-bond interaction play a pivotal role for the enhanced reaction kinetics [21–27]. By using an inverse CeO_2_-Cu_2_O/Cu(111) interface as a bifunctional catalyst, low-temperature conversion of methane to methanol with high selectivity at 450 K in the presence of water was achieved [28,29]. On such a heterogeneous interface, hydroxyl groups formed by water dissociation can directly interact with CH_3_* species to yield methanol with high selectivity, and further decomposition of CH_x_ intermediates to CO and CO_2_ was blocked. The concurrent activation of water and methane at the heterogeneous interface likely underwent a completely new reaction route and synergetic reaction mechanism.
  Currently, the enhanced reaction kinetics of water activation is mainly attributed to H-bond interactions between water reactants and/or reaction intermediates at the nanoscale interface. However, the H-bond interaction is too weak for the dissociation of water. In addition, the catalyst design mainly focuses on the optimization of the size, morphology, composition, valence state, *etc.* of metal components, neglecting the influence of surface adsorbed species and/or their interaction on catalytic performance. Recently, we demonstrated that, when structural water molecules (SWs) are adsorbed at the nanoscale interface or confined nanospace in the form of hydrous hydroxide complex (OH^−^ · H_2_O) and/or water dimmer, a p band intermediate (or transient) state (PBIS) could be formed by space interaction due to the overlap of p orbitals of two O atoms in SWs (Fig. 8a and c) [30–33]. This interaction provides an alternative channel for surface energy and/or electron transfer. Here, the acting mode of SWs is completely different from that of H-bonded water, which is evidenced by a panel of optical spectra with a feature of delocalized π bonding. The concept of SWs not only answers a century of debate whether water is a photoluminescence emission center or not [34,35], but also provides new insights to understand microkinetics of heterogeneous catalysis at the molecular level (Fig. 8b) [36–40]. Based on the molecular orbital theory (MO), the essence of the PBIS can be simply understood as that, due to the spatial overlap of the orbital of the adsorbed atoms (or molecules), two pairs of local electrons in surface chemical bonds can be highly delocalized into four interfacial bonding regions (Fig. 8c), like ‘electron pool’ at the nanoscale interface (*here, localized electrons must invariably be transformed to a collective-electron description, or called ‘interface conjugation or delocalization’*) [31]. The physical nature of PBIS is exactly the same as the transition state (TS) of the reaction intermediate, where the old bond is not completely broken and the new bond is not completely formed.
  With this model, the real catalytic active site of HER for water splitting was identified as structural water molecules (SWs) adsorbed on metal centers in the form of (OH_ad_ · H_2_O@M^+^) (Fig. 8b), where M^+^ can be transition metal ions, or even alkaline metal (AM) cations, but M^+^ is not necessary [40]. This answers that even metal-free carbon nanostructured materials by heteroatom doping (O/N/S/P) can be highly efficient HER catalysts [41–43]. The reaction kinetics of HER with two electrons is well-understood. Compared with the HER, oxygen evolution reaction (OER) with multiple-step electron transfer (4e transfer process) and the formation of multiple intermediates of adsorbed O species (*OH, *OO and *OOH) is more complicated, but plays a more important role in various electrochemical energy conversion processes, such as water splitting, fuel cells and metal air cells. Due to the limitation of scaling relation of adsorbed O species, the reaction kinetics of OER is sluggish, and efficient OER electrocatalysts with low cost are needed to promote its four-electron transfer. At present, the activity of OER is considered highly dependent on the high valence of metal sites. Transition metal sites with high valence can accelerate the reaction kinetics by optimizing the binding strength of adsorbed active species, providing high intrinsic activity. Interestingly, our recently proposed p band intermediate state model not only emphasizes the key role of metal site on the adsorption strength of surface species (reactant, intermediate and product), but also concentrates on the pivotal role of interfacial electron delocalization of surface species (including surface doped heteroatoms) on the electron and proton transfer (Fig. 8b), which could break the scaling relationship of adsorbed O species, thus boosting oxygen evolution. Very recently, by using the same model, we clearly explained the decisive role of water dissociation at the nanoscale interface in determining the catalytic reduction rate of 4-nitrophenol (4-NP) to 4-aminophenol (4-AP) by hydride [36,38]. The hydrogen source of the final product 4-AP came from water instead of NaBH_4_ [44,45]. We may conclude that SWs at the confined nanoscale surface, on the one hand, provides an alternative channel for proton coupled electron transfer (PCET) by interfacial delocalization; on the other hand, it concomitantly acts as an anchoring point to stabilize the transition state thus accelerating the reaction kinetics.
- **Redox chemistry.** The core of green carbon science is essentially redox chemistry, where oxidation and reduction are basically the two opposite and unified halves of the carbon energy system in the carbon cycle (Fig. 1). In nature, redox chemistry as a fundamental principle sustains the energetic foundation of the living system by biocatalysis: the photosynthesis of plants (6CO_2_ + 6H_2_O + solar energy → C_6_H_12_O_6_ + 6O_2_) and the respiration of animals and humans (C_6_H_12_O_6_ + 6O_2_ → 6CO_2_ + 6H_2_O). Hence the carbon balance was well maintained before the Industrial Revolution. For modern society, redox chemistry further plays a role in chemical transformation in the carbon cycle as well as in the construction of a circular economy (Fig. 1).
  Redox reactions are reactions in which changes occur in the oxidation numbers of atoms in involved species. In other words, oxidation and reduction are, respectively, the consequences of loss and acquisition of electrons. Catalysts are often involved in redox reactions and catalytic active sites directly participate in the electron transfer processes. Several typical results in thermal catalysis, electrocatalysis and photocatalysis are exemplified here to show the catalyst participating or promoting electron transfer processes.
  The widely used key catalysts for the chemical transformation of ternary molecular system CO_2_/H_2_O/CH_4_ to high-value fuels are metal oxides with reversible reduction characteristics (M_x_O_y_ ↔ M_x_O_y-1_ + 1/2O_2_), where the redox chemistries of multivalent metal oxides or metal oxide composites play a pivotal role in mediating the interfacial electron transfer. Using multivalent metal or metal oxide catalysts, the development, evolution and current status of solar energy–aided syngas production via redox pair-based water/carbon dioxide splitting thermochemical cycles have been recently reviewed [46]. The ‘proof-of-concept’ of solar energy–driven syngas production was demonstrated, but searching for metal oxide catalysts with high lattice-oxygen carrying/storage capacity and high activity is urgently needed. Very recently, adapting the chemical looping (CL) concept, an updated Na_2_WO_4_ catalyst by decorating an oxygen carrier FeMnO_3_ in oxidative coupling of methane (OCM) exhibits an excellent catalytic performance at 800^o^C, achieving a high space time yield of 29.8g_C2-C3_ · kg_cat_^ · −1^ · h^−1^ with 20% CH_4_ conversion and 80% C_2_-C_3_ selectivity and a low catalyst/CH_4_ weight ratio of 13.5. The used catalysts can be regenerated by a separated oxidation process. Scaled up CL-OCM testing with 10 g catalyst yields comparable results seen in the case of using 1 g of catalyst with good cycling performance, validating the great application potential of the CL-OCM process [47].
  Electrocatalysis, by manipulating redox chemistry at the electrode surface using renewable energy, has been regarded as a clean and promising approach for realizing the low carbon circular economy. The key of electrocatalysts is to break the limitation of unfavorable scaling relationships for binding energies of reaction intermediates by surface engineering and nanoconfinement effects [48,49]. So far, diversified multifunctional electrocatalysts, including metals, transition metal oxides, perovskite oxides and metal chalcogenides have been developed for boosting the kinetics of water splitting (HER and OER), ORR and CO_2_RR, *etc*. Based on the calculation of DFT theory, Nørskov *et al.* [49] suggested a number of alternative strategies to design highly effective electronanocatalysts by bi- or multi-functionalization, promoter addition, alloying, heteroatom doping (O/N/S/P), electrolyte engineering, tethering of organic moieties or complexes and nanoconfinement, *etc*.
  Interestingly, note that, in some cases, redox chemistry occurs with long-range electron transfer at the nanoscale range, often coupling with long-range proton transfer, for example, water splitting dominated by enzyme catalysis (photosynthesis system two, PS II). PSII has a unique cubane structure of a Mn_4_CaO_4_-cluster confined in a unique microenvironment as an active site for water activation [50]. But, the micro-kinetics for water splitting at confined nanospaces is not clear. Using the PBIS model (Fig. 8) [31–33], we proposed that, the structural water molecules (SWs) adsorbed on the cubane cluster play a main role in mediating synergetic electron and proton transfer. When two centers of oxidation and reduction are far away, for example, the long-range electron and proton transfer between PSII and PSI could occur by water bridge or water line in the enzyme pocket, where two more water molecules are linked together by p orbital overlaps [40]. Based on both the principle of our SWs model and the knowledge of ‘biological water’, the design of bio-inspired redox catalysts needs the precise control of water structures near the catalytic sites in three dimensions, breaking away from conventional two-dimensional catalysts with a focus of active centers.
- **Engineering thermochemistry.** Engineering thermochemistry can be regarded as implementing thermochemical transformation by engineering means. Thermochemical transformation is an important approach for accomplishing molecular bond breaking and reconstruction. The mutual complementation of thermal processing and catalytic processing plays an important role in the development of the modern energy industry. For establishing a circular economy, the significance of engineering thermochemistry is obvious, either in the efficiency of large-scale conversion or in the effectiveness of large-scale industrialization. Process efficiency here refers to both energy efficiency and carbon efficiency.
  Thermochemical conversion processes can be divided into three categories: incineration, gasification, and pyrolysis [51], according to the degree of oxygen involved, corresponding, respectively, to fully oxidized, partially oxidized, and non-oxidized. Among them, pyrolysis process usually displays relatively high energy efficiency, low pollution, and low cost in providing fuels and chemicals. Thermochemical conversion processes are usually characterized by free radical mechanisms. The radical is highly reactive and thus short-lived, with a paramagnetic spin magnetic moment for its unpaired electrons. The radicals have a large degree of freedom in the gas and liquid phases, and thermal energy helps to increase that degree of freedom. Reactions can occur between gas-phase radicals and solid-phase surface species. In liquid phase, molecular enrichment greatly promotes bimolecular reactions such as oligomerization, condensation, and coking.
  For carbon-based energy industry, thermal processing has been used extensively, such as gasification of coal and municipal waste, coking of residue oil, steam cracking of naphtha, pyrolysis of biomass and waste plastics, *etc*. Some specific features in process functions of thermochemical conversion are prominent, such as decarbonization, deep cracking, and solids handling and processing. These features make thermochemical processes almost irreplaceable in many aspects. Decarbonization is commonly used in refineries for processing highly hydrogen-deficient feedstocks, such as heavy crude oil and residual oil. Deep catalytic cracking technology was developed at the end of the last century based on FCC technology for producing small molecule olefins, especially propylene. The conversion of solid raw materials is often embodied in the transformation of the physical phase, with products in liquid and gas phases. For processing of a huge volume of solid materials with severe and comprehensive molecular cleavage and reconstruction, it is unimaginable without highly effective thermal processing methods. A top priority is to develop highly efficient thermal conversion processes with high energy and carbon efficiency by incorporating carbon capture and utilization facilities.
  As shown in Fig. 5, the carbon energy study is now increasingly focused on the small molecules such as carbon dioxide, water and hydrogen. Since the molecules of carbon dioxide and water are inherently end products of oxidation in the process of energy acquisition, they have exceptional stability. In fact, all that we are now doing for carbon neutrality is to conduct a repeated cycle: bond breakage and reconstruction of carbon dioxide and water. Of course, energy supplement is necessary and the preferred energy is solar energy. Various means are being tried for the purpose: catalysis, electrolysis, photolysis, photo-electrolysis, biolysis, *etc*. Another important small molecule is methane. It is rich in hydrogen and also highly stable. Facing the above challenges, engineering thermochemical approaches are expected to play a role in solving these problems.
  It is acknowledged that thermal energy intensifies chemical bond vibrations triggering bond breakage. Microwave electromagnetic radiation can not only assist heating and improve heating uniformity, but also reduce the activation energy barrier of transition states in some reactions [52]. Plasma is formed by the bombardment of chemical bonds by high-energy electrons, and the activation energy barrier for a reaction can be overcome through the formation of free radicals and ions [53]. Plasma catalytic systems, in which the plasma and catalyst interact with each other, often form a synergistic effect that is highly beneficial to the target reaction [54]. It can be considered that both microwave and plasma have played an important role in promoting the development of engineering thermochemistry, especially in promoting technological innovation, and it is expected to see the emergence of novel thermochemical processes in the coming years. For instance, high conversions of methane and CO_2_ to valuable syngas at near-ambient temperatures were achieved by using low-power radio frequency inductively coupled plasma (RF-ICP) [55]. Moreover, CO_2_ hydrogenation towards methanol in good yield has also been realized by this strategy in the presence of a Co_x_O_y_/MgO catalyst [56].
- **Dynamic behavior at the nanoscale.** Heterogeneous catalysis is a sophisticated multi-step process. What really happened to the molecules and their courses in pores, especially in nanoscale pores of the catalyst, is by far the most important and least understood phenomenon. All the redox active sites are dynamic. However, for porous catalysts, the diffusional effects in zeolite cavities or cages have been paid much attention in recent years. The more frequently used term for zeolite catalysts is diffusional restrictions. The evolvement of the transfer-related concepts is in progress: size exclusion [57], intracrystalline diffusional resistance [58], shape selectivity [59], confinement effect [60], *etc*. On the other hand, the zeolite catalysts function in another dimension: acidity, basicity, oxidation ability, hydrocarbon pool formation, and so forth. The object of our quest to gain further understanding is essentially the system constituted by the reactions and their environment as a whole. For a catalytic reaction in nanoscale pores, the nanopore provides a so-called microenvironment, which may imply active sites in confined spaces. Therefore, the term nanoreactor may conform more exactly to the reality we studied, which holds the whole scene including host zeolite pores with active sites, and guest molecules, together.
  The study of molecular behaviors in a nanoreactor obviously covers two aspects, according to its literal meaning, nanoscale and dynamic characteristics. The development of zeolite catalysis in the area of heterogeneous catalysis is a milestone in progress. The pivotal factor can be attributed to nanoreactors provided by zeolites with different pore structures and activity characteristics. The supercage in Y zeolite, as a nanoreactor, plays a role in promoting bi-molecular reactions [61]. Hence, gasoline yield and quality are greatly enhanced in catalytic cracking processes. The confined space in the ZSM-5 pore channel constitutes a super nanoreactor for xylene isomerization [62], by accommodating the preferred reaction intermediate to achieve ultra-high selectivity to para-xylene production. The titanosilicalite zeolite TS-1 is characterized by the existence of framework titanium atoms, providing active sites with oxidation performance. The special nanoreactor of TS-1, as a general recognition, is the zeolite pore channel combined with framework Ti active sites. For MTO processes, the catalysts used are mainly SAPO-34 and ZSM-5 [63]. The very tiny nanosized cage of SAPO-34 served as a special nanoreactor for methylation of the pre-formed aromatic species. The highly active methylation reaction in the confined space is the thread running through the sequential reaction scheme in MTO processes.
  Figure 9 illustrates the functionalities of the nanoreactor of SAPO-34. With both acidity and cavity, a series of reactions occur in the nanoreactor. Starting from the adsorption state of surface methoxy species (SMS), initial C–C bonds formation triggers aromatic species formation. The unique behavior of the SAPO-34 nanoreactor is the extremely active methylation of aromatic species in the ultra-confined space, leading to the formation of polymethylbenzenes, which is the origin of low carbon olefin products. Also, due to the repeated methylation, a paralleled reaction in the same nanoreactor is the formation of fused-ring aromatic species, which play roles as coke precursor finally leading to coke deposition. The coke formation leads to the quick deactivation of these nanoreactors. From birth to death, it is a rather short journey for the nanoreactor of SAPO-34. Nevertheless, a series sequential and complicated reaction steps are completed in the same confined space.
  Besides the repeated methylation reaction and olefin production, bimolecular hydrogen transfer reactions between hydrocarbon molecules originating from poly-methyl aromatics might co-exist. However, olefin chain growth, olefin oligomerization, and hydride transfer processes scarcely occur in the nanoreactor of SAPO-34 [63]. The chabazite CHA structure of SAPO-34 possesses cages (∼1 nm) and much smaller 8-atom ring windows (∼0.38 nm) between interconnected cages. Hence, aromatic species can be formed in the cage but cannot be transferred through the windows. Therefore, a unique nanoreactor with constrained aromatic species and freely entering methanol molecules is formed to attain a high level of methylation. Olefin production in SAPO-34 nanoreactor proceeds by paring or side-chain mechanism based on polymethylbenzene intermediates. In comparison, the MFI structure of ZSM-5 possesses two-dimensional channel and 10-atom ring windows. The channels are wide enough for the transfer of molecules such as tetramethylbenzene. The channel intersections of ZSM-5 provide enough volume for cyclization reactions and bi-molecular hydride transfer reactions which convert olefins into alkanes and aromatics [63]. Only in the case of zeolites with appropriate channel structure such as MFI, the aromatic cycle and olefin cycle can be balanced and adjusted [63].
  However, one of the critical factors is the dynamic behavior of molecules in the nanoreactor of zeolites, which is often a decisive factor in determining different reaction pathway selectivity for the total reaction scheme. The confinement space of the nanoreactor causes *immobilization* of different molecules or species to a different extent and bringing different impacts on reaction pathways. In the case of SAPO-34, the aromatic species in the nanoreactor is actually *trapped* immediately upon formation. The unique reaction condition is the co-existence of *trapped* aromatics together with methanol and olefin molecules having freedom in going in and out of the nanoreactor because of their smaller size. The *trapped* aromatic species become the target of methylation reactions. In the case of ZSM-5, the aromatic and olefin molecules are only *retarded* by nanosize channels rather than *trapped*, the mobility of aromatic and olefin molecules is significantly higher hence the number of methyl substitution on the aromatic ring is lower. Also, due to the molecular mobility, the reaction pathway selectivity to the bimolecular hydrogen transfer is much higher, leading to the formation of higher carbon numbers of hydrocarbons.
  The significance of dynamic behavior of molecules toward reactions in the nanoreactor can also be observed by changing diffusion path length and contact time. In the competition of aromatic cycle and olefin cycle mechanisms, ethylene and total olefin selectivity can be used as an indication of higher propagation of the aromatic cycle. It is proved that ethylene and total olefin selectivity increased with the increase of crystallite size [64,65] and contact time [66] for MFI and MEL zeolites in MTO reactions. The higher propagation of aromatic cycle is attributed to the increase of intra-crystallite residence time and the promotion of methylation reactions in the nanoreactor. The impact of dynamic behavior on mechanistic selectivity at the nanoscale is also evidenced by changing the morphology and/or pore structure of zeolites in a number of studies, such as nanosize [67], nanosheet [68,69], mesoporous structure [70], hierarchically pore structure [71], *etc*.
- **CO_2_ reaction engineering.** Carbon dioxide is the end product of carbon energy utilization and is also the most important greenhouse gas. CO_2_ conversion and utilization are a must to address carbon neutrality, meanwhile, it is a great challenge to make use of the fully oxidized end product as a starting carbon material for fuel and chemical production. The research effort is highly active and extensive in this area and numerical results related to novel scientific and technical findings have been reported in recent years. However, global CO_2_ emission is continuously increasing year by year. The large and concentrated amount of emissions are, in many cases, attributed to large-scale industry. Obviously, the development of industrial-scale solutions for carbon capture, utilization, and storage is the most important and practical meaning. Corresponding to *coal industry, gas industry*, and *oil industry*, it is now urgent to develop *CO_2_ industry* for CO_2_ transformation, given the crisis of the unceasing rise of atmospheric temperature.
  Most of the heavy CO_2_ emitters such as refineries, steel and cement manufacturers, *etc.*, have the infrastructure in place to implement CO_2_ separation and processing technologies. The immediate and effective solution to deal with flue gases in heavy carbon industries is to enforce technologies for CO_2_ valorization such as methanol production, dry reforming, methanation, or RWGS, although modification on the basis of low carbon and low cost is necessary. Process engineering is important in carbon capturing, reactor engineering, process integration and intensification. Based on the ternary elemental chemistry of carbon, hydrogen and oxygen and related ternary molecular systems in Figs 2 and 3, different strategies for process integration or combination could be decided according to the practical circumstance of the local plant. Figure 3 has illustrated that process development can be accomplished by reaction or process combination, such as BRM, TRM, Carnol process, *etc.* A demonstration unit of CO_2_ hydrogenation for methanol production at a scale of 1500 Mt/a has been operating in Lanzhou, China, since 2021. A novel solid solution catalyst was used with a high stability of more than 500 h on stream and a methanol selectivity in the range of 86%–91% [72]. The next stage target of the project is to integrate CO_2_ hydrogenation with solar-driven water splitting, rather than using hydrogen from water electrolysis as the present stage. A homogeneous reaction process using metal complexes as a catalyst has also been developed successfully for CO_2_ hydrogenation to produce formate and methanol, which is directly integrated with CO_2_ capture by aqueous amine and hydroxide solutions [73].
  The introduction of micro-channel reactors often makes remarkable progress for the chemical processes in the industry in many ways such as process intensification, efficiency, productivity and safety [74]. Biphasic reactions are generally accelerated using flowing microreactor compared to batch processes due to the significant increase in surface areas between the layers and the increased mixing within each liquid slug [75]. Owing to the unusual heat and mass transfer performance, micro-channel reactors are proven to be effective for engineering CO_2_ capture and utilization, affording solutions to control the multiphase contact based on high interfacial area [76,77].
  The usage of microscale reactors with high surface area to volume ratio significantly enhanced the CO_2_ absorption efficiency into amine solvents. Under certain operating conditions, close to 100% CO_2_ absorption efficiency was observed for absorption of CO_2_/N_2_ mixture into aqueous diethanolamine [78]. For intensified dimethyl ether (DME) production from syngas with CO_2_, the hot-spots usually observed in packed-bed units was suppressed by more than 75% by using a wall-coated and air cooled microreactor [79], leading to almost double space time yields of DME. For CO_2_ methanation, a microchannel reactor exhibited good CO_2_ conversion (83.4%) and high CH_4_ productivity (16.9 L · g_cat_^−1^.h^−1^) [80]. Moreover, such a microchannel reactor demonstrated satisfactory durability at demanding operation conditions with high space velocity and high temperature.
  Membrane technologies are extremely attractive in providing solutions for CO_2_ capture and also for its conversion toward value-added chemicals [81]. A CO_2_-permselective membrane reactor was studied experimentally and analyzed by a mathematical model for SMR, aiming at hydrogen production with simultaneous CO_2_ removal [82]. The results showed that the membrane reactor provided over 99% CH_4_ conversion, H_2_ yield, and CO_2_ recovery and produced an essentially pure H_2_ stream with zero CO concentration. For CO_2_/CH_4_ separation, single channel tubular CHA membranes displayed excellent selectivity for separation of industrially relevant CO_2_/CH_4_/H_2_O mixtures [83]. The key component of the membrane were well-intergrown and smooth CHA films with thickness <500 nm, which provided selective interaction between CO_2_ and H_2_O within the pores. The highest observed CO_2/_CH_4_ separation selectivity could reach 198 at a feed pressure of 600 kPa (including 2.2 kPa water) and 293 K. These results illustrated that the membranes are promising candidates for industrial separation of CO_2_ from, for example, natural gas and biogas. With the purpose of commercial application, Air Products and its partner, Ceramatec, have developed ion transport membranes for the production of synthesis gas [7,84]. The ion transport ceramic membranes can combine the unit operations of air separation and autothermal reforming into a single unit operation. Economic analyses of the syngas process showed that the capital cost for syngas generation could reduce by up to 30%. The membranes were tested in a 27 Nm^3^/h pilot scale facility at syngas pressures up to 3 × 10^6^ Pa (absolute) and temperatures up to 1323 K.

The implicit concern of the expression ‘green carbon science’ points directly to the goal of carbon neutrality and the reconfiguration of energy systems. In the discussion of the fundamentals of green carbon science, it seems that a ‘molecular vista’ becoming clearer for the construction of a new energy system, by effectively choosing and stacking building blocks such as chemical elements, atoms, molecules, chemical bonds, bonding electrons, *etc*., B. M. Weckhuysen *et al.* [85] said that, ‘a new era is in its infancy: one that will be defined by pasting small carbon molecules, rather than cutting large ones (for example, crude oil); one that will be shaped by recycling, electricity and renewable hydrogen’.

Carbon energy has contributed to the creation of human civilization, and it can be considered that the configuration of the carbon energy system is one of the important laws that govern the operation of everything in the universe. The core of the carbon energy system is the opposition and unity of two aspects: oxidation and reduction. The operation of oxidation and reduction is based on the ternary elemental system composed of the three elements of carbon, hydrogen and oxygen.

Four Chinese characters ‘Da Dao Zhi Jian’ (大道至简), are often used in summarizing the understanding of Lao Zi’s philosophy. It interprets as ‘The greatest truths are the simplest’.
